# Changes in self-confidence in professional, personal, and scientific skills by gender during physician-scientist training at the University of Pittsburgh

**DOI:** 10.1017/cts.2024.556

**Published:** 2024-06-05

**Authors:** Tumader Khouja, Chelsea N. Proulx, S. Mehdi Nouraie, Ashti M. Shah, Rashmi J. Rao, Richard A. Steinman

**Affiliations:** 1 Department of Community Dentistry and Behavioral Science, University of Florida, Gainesville, FL, USA; 2 Clinical and Translational Science Institute, University of Pittsburgh School of Medicine, Pittsburgh, PA, USA; 3 Division of Pulmonary, Allergy, and Critical Medicine, Department of Medicine, University of Pittsburgh School of Medicine, Pittsburgh, PA, USA; 4 Physician Scientist Training Program, University of Pittsburgh School of Medicine, Pittsburgh, PA, USA; 5 Department of Medicine, Hematology/Oncology, University of Pittsburgh School of Medicine, Pittsburgh, PA, USA; 6 Department of Pharmacology & Chemical Biology, University of Pittsburgh School of Medicine, Pittsburgh, PA, USA

**Keywords:** Physician scientist, women, Medical Scientist Training Program, Physician Scientist Training Program, residents, fellows, self-efficacy, confidence, curriculum, gender

## Abstract

**Introduction:**

Persistence in physician-scientist careers has been suboptimal, particularly among women. There is a gender gap in self-confidence in medicine. We measured the impact of our physician-scientist training programs on trainee’s confidence in professional, personal, and scientific competencies, using a survey measuring self-rated confidence in 36 competencies across two timepoints.

**Methods:**

Results were analyzed for the full survey and for thematic subscales identified through exploratory factor analysis (EFA). A mixed effects linear model and a difference in differences (DID) design were used to assess the differential impact of the programing by gender and career level.

**Results:**

Analysis included 100 MD-PhD or MD-only medical student or resident/fellow trainees enrolled between 2020 and 2023. Five subscales were identified through EFA; career sustainability, science productivity, grant management, goal setting, and goal alignment (Cronbach’s alpha 0.85–0.94). Overall, mean scores increased significantly for all five subscales. Women significantly increased their confidence levels in all five areas, whereas men increased only in science productivity and grant management. Mixed effects models showed significant increases over time for women compared to men in career sustainability and goal alignment. Residents and fellows had greater increases than medical students across all subscales.

**Conclusion:**

Physician-scientist trainees fellows increased their confidence in personal, professional, and scientific skills during training. Training had a greater impact on women than men in building confidence in sustaining careers and aligning their goals with professional and institutional priorities. The magnitude of increased confidence among residents and fellows exceeded that in medical students.

## Introduction

The growing promise and burgeoning complexity of biomedicine warrant a robust physician-scientist workforce. However, attrition in the physician-scientist path has been a longstanding problem [1–7]. Sustaining a physician-scientist career not only requires investigative and clinical skills but also versatility in navigating competing time commitments, sustaining innovation and funding, and prioritizing clinical, research, and personal goals [8].

Women face unique difficulties navigating physician-scientist careers compared to men. Women comprise a minority of funded investigators [9,10] apply for grant funding at a lower rate and are cited less often [4]. Women MD-PhD’s who attained NIH predoctoral grants are only 37% as likely as their male counterparts to eventually have independent NIH research funding [11].

Women express lower confidence in career advancement in medicine [12,13], and in knowledge and performance despite equal clinical knowledge and skills as men [14]. Women residents and fellows participating in a clinical research training program scored lower than men when self-assessing their ability to conduct clinical research [15]. While systemic factors contribute to the shortfall in physician scientists and disproportionately affect women [16–21], gender gaps in confidence and self-efficacy could counter the resiliency needed to overcome barriers to success for physician scientists.

The Clinical Research Appraisal Inventory (CRAI) has been used to measure confidence in research skills [22,23]. The CRAI however, focuses on the research domains pertinent to conducting clinical research studies. No investigation to date has measured confidence in research, career, and personal domains in a physician-scientist cohort focused on basic science and laboratory-based translational research.

The University of Pittsburgh School of Medicine training portfolio includes the Medical Scientist Training Program (MSTP, MD-PhD) and Physician Scientist Training Program (PSTP) [24,25] medical student programs and a Burroughs Wellcome Foundation supported Physician Scientist Incubator Program that trains MD-only residents and fellows in preclinical research. While PSTP is an acronym also used for resident and fellow training programs, our PSTP is specific to medical students as described by Steinman et al. [24]. We refer herein to participating medical students as “MSTP/PSTP” and to the resident/fellows in the BWF Incubator program as “BWF Fellows.”

We developed a Physician Scientist Confidence questionnaire to measure self-confidence in scientific, personal, and professional competencies at early and later points in the training process in these three programs. Our objective was to evaluate the programmatic impact on trainee’s confidence over time and by gender. A secondary objective was to assess the impact of training on confidence rankings by career level.

## Results

### Cohort characteristics

There were 102 trainees who completed the survey at the two time points administered through Research Electronic Data Capture (REDCAP) [26,27]. Two individuals were not included in the final analysis – one individual preferred not to identify gender; another individual had logged back into the time 1 survey at time 2. The cohort included 61% female trainees. 82% were enrolled in the MSTP/PSTP program and 18% in the BWF incubator program. Full demographics of the sample are shown in Supplement, Suppl. Table 1. The average time between initial and follow-up survey responses was 1.6 years (see detail in Supplement, Suppl. Methods). All participants included in analyses consented under an expedited protocol approved by the University of Pittsburgh Institutional Review Board. 57% of consented eligible individuals completed both surveys. There was no significant in age or gender distribution between those who completed both surveys and are included in this analysis and those who are not included because they answered only 1 or neither survey, did not respond, or declined consent (see Supplemental Methods, p.S23).

### Difference in responses to individual survey questions

Overall, mean scores across all confidence survey items increased at follow-up by a mean of 0.64 (95% CI, 0.25–1.03) on the 11-point scale.

**Figure 1.**
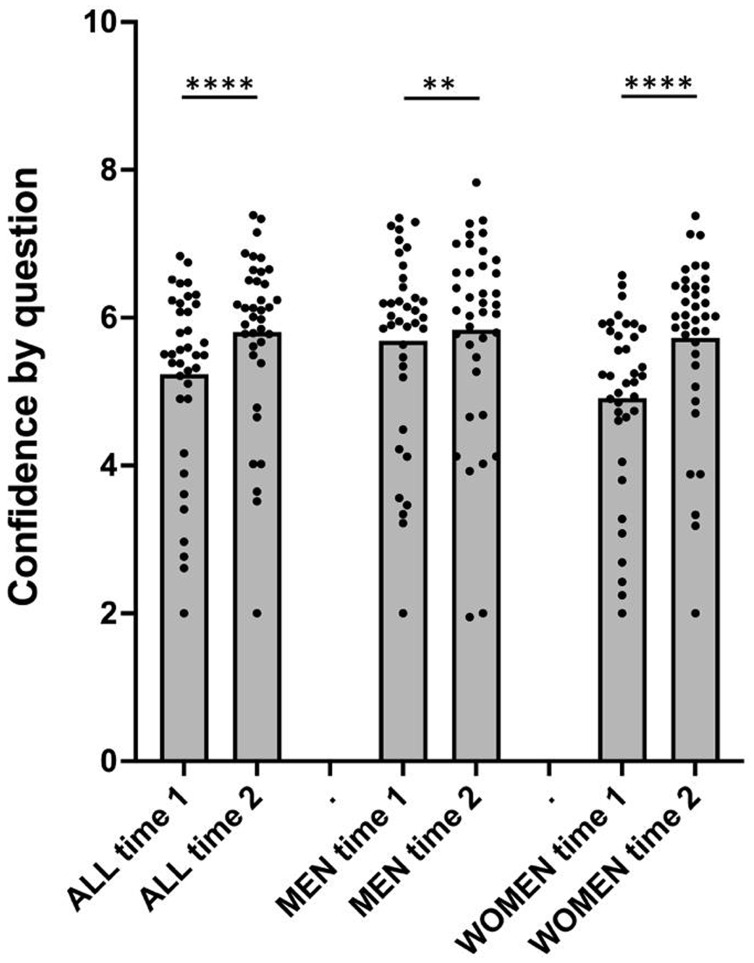
Mean responses to confidence ranking items in total, by gender and by survey time. Mean responses by 100 trainees to each of 36 ranking items are shown. Each dot represents an item on the survey. Bars show mean of indicated group/time to all 36 ranking items. **p < 0.01, ****p < 0.0001, by paired (t1 v t2) *t*-test by group.

Figure 1 shows average level of confidence by response to each individual item in the survey for the total cohort, men and women. Responses to survey items by training level are shown in Supplement, Suppl. Figure 1. Overall, confidence increased over time. While both men and women rated their level of confidence higher at time 2, this increase was more marked for women. Averaging all item responses, women rated their level of confidence lower than men at time 1 but not at time 2.

For the entire cohort, mean confidence scores increased for 35 of 36 items, with a small decrease (0.153, 2.3% change from initial level) only in confidence in the ability to “*Nourish your physical and emotional health.”* This decrease was seen in the response of both women and men. Women increased their confidence in response to all other (35/36) items, whereas men rated their confidence higher for 27 and lower for 9 items (see Supplement, Suppl. Figure 2). At time 2 (compared to time 1), the average increase in confidence scores by women rose by 0.56 (95% CI 0.045 to 1.07) more than the increase in men’s scores.

### Grouping of survey competencies and mean scores across subscales

To identify thematic subscales, we conducted an exploratory factor analysis. Exploratory factor analysis (EFA) analysis identified five subscales: *Career Sustainability, Science Productivity, Grant Management, Goal Setting, and Goal Alignment,* shown along with the contributing ranking items in Table 1.

**Table 1.** Listing of subscales identified through exploratory factor analysis and the survey items grouped within each subscale

Subscale	Survey Item for ranking of confidence level
Career sustainability	Find a job that aligns with your skills.
	Find a job that aligns with your career goals or career path.
	Effectively market your skills during the job search and application process.
	Initiate research collaborations with colleagues.
	Sustain effective collaborations.
	Terminate a collaboration that isn’t working.
	Finding opportunities to network with individuals in my field.
	Using networking strategies to establish relationships with individuals in my field.
	Orally present results at a regional or national meeting.
Science productivity	Determine an adequate number of subjects/animals/repeats for your research project.
	Write the results section of a research paper that clearly summarizes and describes the results, free of interpretative comments.
	Write a discussion section for a research paper that articulates the importance of your findings relative to other studies in the field.
	Select a suitable topic area for study.
	Identify faculty collaborators from within and outside the discipline who can offer guidance to the project.
	Frame a testable hypothesis related to but independent of your mentor’s hypothesis.
	Give a compelling elevator pitch summarizing a research project.
	Assess whether an opportunity offers personal growth.
	Assess whether an opportunity offers professional growth.
	Write a literature review that critically synthesizes the literature relevant to your own research question.
	Obtain reagents, tissue samples, and/or databases for research purposes.
Grant management	Draft a compelling specific aims page for a competitive grant.
	Arrange for constructive feedback on a grant proposal draft.
	Describe a major funding agency’s (e.g. NIH, NSF, or foundation) proposal review and award process.
	Prepare a project budget for a grant application.
	Recruit and screen research project staff.
	Manage and supervise research project staff.
	Identify appropriate funding sources (local, state, national) to support a study.
Goal Setting	Develop a daily writing routine.
	Set achievable personal and professional goals along with a plan to meet them.
	Make and use strategies to productively balance research and clinical time commitments.
	Make and use strategies to productively balance academic and nonacademic time commitments.
	Nourish your physical and emotional health.
Goal alignment	Say no to opportunities that do not offer personal growth.
	Say no to opportunities that do not offer professional growth.
	Recognize when your personal values and institutional priorities are aligned.
	Balance your time with institutional priorities and your personal values.

The subscales were compared by training level and gender as summarized in Table 2.

**Table 2.** Mean scores of self-confidence in professional and scientific competencies subscales at time 1 and time 2, overall, by gender and by career level

Subscale	N	Time 1		Time 2		Difference (time 2-time 1)	SD	p-value
		Mean	SD	Mean	SD			
***Overall***								
Career sustainability	97	**5.3**	2.0	**5.9**	1.6	0.6	1.7	<0.001
Science productivity	98	**6.0**	1.5	**6.6**	1.3	0.6	1.3	<0.001
Grant management	98	**3.8**	2.0	**4.8**	1.8	1.0	1.8	<0.001
Goal setting	100	**5.3**	1.8	**5.8**	1.7	0.5	1.6	0.010
Goal alignment	97	**5.7**	1.7	**6.0**	1.6	0.3	1.7	0.048
***Male***								
Career sustainability	38	**6.0**	1.9	**6.2**	1.3	0.2	1.6	0.373
Science productivity	38	**6.5**	1.4	**6.9**	1.1	0.4	1.2	0.081
Grant management	38	**4.2**	2.2	**5.0**	1.6	0.8	1.7	0.009
Goal setting	39	**5.7**	1.7	**5.9**	1.6	0.2	1.5	0.322
Goal alignment	38	**6.2**	1.4	**6.0**	1.5	−0.2	1.6	0.354
***Female***								
Career sustainability	59	**4.8**	1.9	**5.7**	1.8	0.9	1.6	<0.001
Science productivity	60	**5.7**	1.5	**6.5**	1.5	0.8	1.4	<0.001
Grant management	60	**3.5**	1.8	**4.6**	1.9	1.1	1.8	<0.001
Goal setting	61	**5.1**	1.9	**5.7**	1.7	0.6	1.7	0.014
Goal alignment	59	**5.3**	1.7	**6.0**	1.7	0.7	1.7	0.002
***BWF***								
Career sustainability	18	**5.1**	1.6	**7.0**	1.1	1.9	1.3	<0.001
Science productivity	18	**5.3**	1.4	**7.3**	1.2	2.0	1.3	<0.001
Grant management	18	**3.7**	1.8	**6.1**	1.5	2.4	1.7	<0.001
Goal setting	18	**5.3**	1.4	**6.8**	1.3	1.5	1.3	<0.001
Goal alignment	18	**5.7**	1.5	**6.6**	1.7	0.9	1.4	0.010
***MSTP/PSTP***								
Career sustainability	79	**5.3**	2.3	**5.7**	1.6	0.4	1.6	0.040
Science productivity	80	**6.1**	1.5	**6.5**	1.3	0.4	1.1	0.009
Grant management	80	**3.8**	2.1	**4.5**	1.7	0.7	1.7	<0.001
Goal setting	82	**5.4**	1.9	**5.5**	1.7	0.1	1.6	0.295
Goal alignment	79	**5.7**	1.7	**5.9**	1.6	0.2	1.8	0.280

p values were derived from a paired *t*-test. Participants who completed research confidence skill items included in each subscale at time 1 and at follow-up were included in the analysis.

MSTP = Medical Scientist Training Program; PSTP = Physician Scientist Training Program (medical student); BWF = Burroughs Wellcome Foundation (BWF physician-scientist incubator for residents and fellows).

Notably, the level of confidence increased for every subscale for the full cohort. The subscale with the smallest increase was *Goal Alignment*, because of a decrease in confidence in skills assigned to this category among men. This was the sole instance of a drop in confidence for a subscale in any trainee group.

Men only increased confidence in the *Grant Management* subscale. In contrast, women showed an increase in confidence in all five of the thematic subscales. In the supplement, Suppl. Table 2 compares men and women for each subscale at both time points. Initially, women ranked significantly lower in confidence than men in 4 of 5 subscales. At follow-up (time 2) there was no significant difference between men and women in any subscale.

We also examined self-rated confidence by training level. Despite the difference in training level, both BWF Fellows and MSTP/PSTP medical students showed similar levels of confidence in the initial survey (time 1, Table 2). Both groups showed the greatest increase in confidence in skills related to *Grant Management,* and also significantly increased confidence in *Career Sustainability and Scientific Productivity.* Only the BWF Fellows significantly increased confidence in the other two subscales, *Goal Setting* and *Goal Alignment.*

The BWF Fellow and MSTP/PSTP groups each had a majority of women respondents (66 and 61% respectively). To assess whether the increase in confidence among the different cohorts was restricted to the women in the resident/fellow group, we calculated mean scores by career level and gender as shown in the Supplement, Suppl. Table 3.

In the BWF Fellow cohort, both men and women increased their level of confidence in 4/5 subscales (in all but *Goal Alignment*). In contrast, in the MSTP/PSTP student group, the change in confidence over time only increased significantly among women. Women exhibited a significant increase in every subscale except for *Goal Setting.*

The increase in confidence by women during the training period remained for certain subscales after adjustment for initial scores in a mixed effects model. The mixed-effects model showed a differential impact of programing by gender for two of the five subscales, *Goal Alignment* and *Career Sustainability*. The model output is shown in Table 3. The increase among females surpassed the increase among males for “*Career Sustainability”* (time v subscale interaction term = 0.68 [95% CI: 0.03–1.33, *p* = 0.042]) and for “*Goal Alignment*” (time v subscale interaction term = 0.96 [95% CI: 0.33–1.59, *p* = 0.003]). Other subscales did not meet the threshold for significance.

**Table 3.** Linear mixed model output for the differential impact of the program by gender. Estimates were derived from a linear regression model with a random intercept adjusting for each subscale’s initial scores in their respective model

a. Career sustainability			
Variable	Coefficient	95% CI	P-value
Career sustainability score at time 1	0.75	0.68–0.83	<0.001
Female (male reference)	−0.28	−0.62–0.06	0.105
Time 2 (time 1 reference)	0.23	−0.21–0.67	0.297
Gender *time	0.68	0.03–1.33	**0.042**

b. Science productivity			
Variable	Coefficient	95% CI	P-value
Science productivity score at time 1	0.75	0.66–0.84	<0.001
Female (male reference)	−0.21	−0.50–0.08	0.161
Time 2 (time 1 reference)	0.36	−0.04–0.75	0.078
Gender *time	0.48	−0.02–0.98	**0.061**

c. Grant management			
Variable	Coefficient	95% CI	P-value
Grant management score at time 1	0.75	0.65–0.84	<0.001
Female (male reference)	−0.18	−0.54–0.17	0.171
Time 2 (time 1 reference)	0.79	0.22–1.35	0.007
Gender *time	0.38	−0.31–1.06	**0.279**

d. Goal setting			
Variable	Coefficient	95% CI	P-value
Goal setting score at time 1	0.76	0.68–0.84	<0.001
Female (male reference)	−0.12	−0.37–0.12	0.325
Time 2 (time 1 reference)	0.24	−0.22–0.70	0.308
Gender *time	0.31	−0.26–0.87	**0.287**

e. Goal alignment			
Goal alignment score at time 1	Coefficient	95% CI	P-value
Initial score of subscale	0.74	0.63–0.86	<0.001
Female (male reference)	−0.24	−0.59–0.10	0.166
Time 2 (time 1 reference)	−0.24	−0.72–0.25	0.341
Gender *time	0.96	0.33–1.59	**0.003**

P values are bolded for the interaction term of program gender and time (Gender*time). This difference in differences estimator is calculated as (Male mean score at time 1- Male mean score at time 2) – (Female mean score at time 1- female mean score at time 2). A *p* < 0.05 indicates a significant interaction term of gender and time.

We also analyzed the effect of the training period by career level in a mixed-effect model. Training had a differential effect by career level across all subscales. This was demonstrated by a significant interaction term between career level and time for each subscale as shown in Table 4. For all subscales, BWF Fellows showed a greater increase in mean scores compared to MSTP/PSTP medical students.

**Table 4.** Linear mixed model output for the differential impact of the program by career level. Estimates were derived from a linear regression model with a random intercept adjusting for each subscale’s initial scores in their respective model

a. Career sustainability			
Variable	Coefficient	95% CI	P-value
Career sustainability score at time 1	0.75	0.66–0.83	<0.001
MSTP/PSTP (BWF reference)	0.055	−0.25–0.36	0.726
Time 2 (time 1 reference)	1.9	1.23–2.50	<0.001
Career level*time	−1.5	−2.19 to –0.80	**<0.001**

b. Science productivity			
Variable	Coefficient	95% CI	P-value
Science productivity score at time 1	0.78	0.70–0.86	<0.001
MSTP/PSTP (BWF reference)	0.18	−0.09–0.44	0.191
Time 2 (time 1 reference)	2.01	1.39–2.63	<0.001
Career level*time	−1.67	−2.34 to –0.99	**<0.001**

c. Grant management			
Variable	Coefficient	95% CI	P-value
Grant management score at time 1	0.75	0.66–0.84	<0.001
MSTP/PSTP (BWF reference)	0.02	−0.35 –0.39	0.932
Time 2 (time 1 reference)	2.38	1.57–3.19	<0.001
Career level*time	−1.67	−2.55 to –0.79	**<0.001**

d. Goal setting			
Variable	Coefficient	95% CI	P-value
Goal setting score at time 1	0.76	0.69- 0.84	<0.001
MSTP/PSTP (BWF reference)	0.02	−0.29–0.34	0.882
Time 2 (time 1 reference)	1.53	0.97–2.10	<0.001
Career level*time	−1.35	−2.00 to –0.69	**<0.001**

e. Goal alignment			
Variable	Coefficient	95% CI	P-value
Goal alignment score at time 1	0.72	0.61–0.83	<0.001
MSTP/PSTP (BWF reference)	−0.01	−0.33–0.31	0.942
Time 2 (time 1 reference)	0.93	0.32–1.55	0.003
Career level*time	−0.72	−1.32 to –0.11	**0.021**

MSTP = Medical Scientist Training Program; PSTP = Physician Scientist Training Program (medical student); BWF = Burroughs Wellcome Foundation (BWF physician-scientist incubator for residents and fellows).

P values are bolded for the interaction term of career level and time (Career level*time). This difference in differences estimator is calculated as (BWF mean score at time1 – BWF mean score at time2) – (MSTP/PSTP mean score at time1- MSTP/PSTP mean score at time 2); *p* < 0.05 is considered significant.

### Lack of change in motivation, satisfaction, or grit

The surveys of self-rated confidence were conducted concurrently with measurement of motivation [28], burnout [29], satisfaction [30], and grit [31]. We explored whether ratings of these measures changed during training. However, no significant changes in motivation, satisfaction, or grit were seen in the full cohort (Supplement Suppl. Table 4, Suppl. Figures 3, 4). Burnout scores increased modestly in the cohort from 1.94 to 2.12 (*p* = 0.03, 95% CI 0.015–0.342). Overall, a relationship between these factors and the observed increase in self-confidence among women was not evident and was not pursued further.

### Curricular element ranking by participants

The medical student PSTP program 24 is a 5-year MD program comprised of 16 months of basic/translational laboratory research in addition to six required PSTP enrichment courses beyond the medical school curriculum; the MSTP MD-PhD program has 9 required MSTP enrichment courses (four co-enrolled by PSTPs) beyond those of medical and graduate school; the BWF Incubator Fellows engage in 2 years of laboratory work concurrent with weekly professional and/or scientific development classes. All three programs share the same director (R.A.S), who instructs the majority of classes. Common training components of all programs include courses or classes on grant writing, whiteboard work-in-progress presentations, directed interviews with near-peer role models, mock study sections, and a variety of classes on professional development topics. All of the programs include career advisors or development committee meetings and 4–6 individual sessions with professional career coaches.

Respondents were asked what curricular features contributed to each subscale by ranking the top 3 out of a list of courses/classes/activities that they felt contributed to each of the five thematic competency subscales (Supplement Suppl. Table 5A). A brief description of each subscale accompanied the list; additionally, text fields were available for comments. Sixty-nine participants (69%) responded to the curriculum survey (8 BWF Fellows, 23 PSTP and 38 MSTP). The top curricular items that were identified in common by all three cohorts for each subscale are shown in Supplement Suppl. Tables 5B, 5C. Professional development classes were linked by all to *Career Sustainability*, whiteboard talks and rigor sessions to *Science Productivity,* and grantwriting classes to *Grant Management.* The 1-on-1 sessions with professional coaches were noted by all cohorts as a top factor in building skills in *Goal Setting* and *Goal Alignment,* consistent with a recent report on coaching for residency transitions [32].

### Other factors that could impact trainee confidence

The BWF Cohort comprises residents and fellows and is older (mean 31.6; median 30.5 years old) than the MSTP/PSTP cohort (mean 25.4; median 25.0) years old. Conceivably being older could position the BWF cohort to benefit more from program elements. However, there was only weak correlation between age and changes in the level of confidence over time for the entire cohort (*r* ^2^ = 0.11, linear regression), men (*r* ^2^ = 0.14), and women (*r* ^2^ = 0.13). Moreover, the BWF cohort did not differ significantly from the medical students (*p* = 0.27) in their ranking of confidence at baseline, despite their age difference.

Mentoring can have a large impact on confidence in physician-scientist skills. All participants were asked, “To what extent do you feel your primary research mentor is meeting your expectations?” From participants as a whole as well as those at each training level and for each gender, the mentors received a median rank of 4.0 (“exceeds expectations”) on a 5-point scale. There was no significant difference between training levels or genders at either time point in participant ranking of mentors.

## Discussion

The objective of this study was to evaluate the impact of our laboratory-linked physician-scientist training programs on trainee’s level of confidence in professional, personal, and scientific competencies over time, by gender and by career level. We observed a significant gender gap in confidence at the initial assessment with females expressing lower confidence in all areas queried. That finding is consistent with reports that women in medicine and science have lower perceived self-efficacy than men [12–14,33]. The onset of this gap in academic confidence is quite early and present in high school if not earlier [34]. This study was the first to explore this gender gap in confidence specifically in pre- and post-doctoral physician-scientist trainees engaged in preclinical research training.

### Increase in women trainee’s confidence

It is striking that the women in this study, whether medical students or residents/fellows, reported an increase in their level of confidence during training. There have been few studies assessing changes in confidence among women in academia. Bakken demonstrated that women training in clinical research ranked their ability in six clinical investigation competencies lower than men; interestingly, men’s confidence increased more than women’s [15] following a skill-building workshop.

Several studies have measured confidence in performance among medical students [35] and medical postgraduates [14]. There was no gender difference among Lerner College of Medicine students in their clinical research confidence (using the CRAI survey) at matriculation or at graduation [36]. Versions of the CRAI have also been used to measure changes in self-efficacy changes following clinical research training or for medical students doing Scholarly Projects; while increases were noted, those studies did not analyze effects by gender [37,38]. The CRAI instrument analyzes confidence in research activities related to design, reporting, conceptualizing, planning, funding, and protecting subjects in studies. Literature indicates that the challenges negotiated by physician scientists extend beyond those activities.

Our instrument was designed specifically for physician scientists in training and structured to encompass not only performance-related domains but also questions related to personal and professional persistence, goal setting, and goal alignment. While several of the items in the *Goal Alignment* and *Goal Setting* subscales are important in personal (as well as academic) settings, this study did not comprehensively explore the range of factors involved in the personal agency of physician scientists.

The magnitude of significant changes in confidence rating for subscales ranged from 0.3 to 1.0 overall, from 0.4 to 0.7 among the medical students, and from 0.9 to 2.4 among the resident/fellow cohort. The magnitude of these changes in confidence is comparable with other assessments of changes in efficacy or confidence in college students, STEM trainees, or medical students [38–40]. Ultimately, the significance of our findings will require correlation of self-ranked confidence with career persistence and success.

In our study, men rated their confidence levels higher than women initially. One could posit that men’s higher initial confidence ranking indicates that men are subject to the Dunning Kruger effect [41] and relatively unaware of their shortcomings. However, the moderate range of men’s initial rankings (from 4.2 to 6.5 out of 10 highest score) suggests that Dunning Kruger overconfidence was not a major factor.

The confidence level scores between men and women were significantly different initially, with women rating themselves lower than men initially but not at follow-up. To compare the change over time in confidence as a function of gender, we used a mixed model correcting for gender differences at the initial assessment. The differential effects of programing by gender were significant for the subscales *Career Sustainability* and *Goal Alignment* after correction for initial scores. Given the evidence that fewer women persist in physician-scientist careers [9,42] it is promising that women in our cohort increased their confidence in these subscales linked to persistence.

### Greater increase in confidence ranking at the resident/fellow level

A secondary objective was to assess differences in self-confidence in professional, personal, and scientific competencies over time by career level. Despite having similar scores initially, BWF Fellows increased confidence across all subscales compared to MSTP/PSTP students. This could indicate that physician-scientist training programs are most effective during residency/fellowship or may be a function of the MD-only BWF Fellow cohort (56% in surgery or surgical specialties) or of our BWF Incubator program curriculum. While similar research and professional competencies were taught in the pre- and postgraduate programs, the context and case studies were tailored to training stage.

### Ratings of motivation, grit, satisfaction, burnout

In addition to our confidence rating questions, we surveyed participants with validated scales for motivation [28], burnout [29], satisfaction [30] and grit [31]. Only burnout scores increased between initial assessment and follow-up, increasing (0.18 on a 5-point scale) in the full cohort and in men but not women. Whether this contributed to the more modest increase in confidence in men compared with women is unclear.

Women scored higher on the grit scale than men both at initial assessment and at follow-up, without a significant change between timepoints. Higher levels of grit may characterize the population of women choosing this long and challenging career path. It is interesting that at time 1, women ranked 9.5% higher than men in grit and yet rated their confidence lower. The linkage between grit and self-efficacy is complex [43], and a career development model proposing interrelatedness of grit and confidence may be insufficient. It remains to be seen whether the confidence scale we employed is a more robust measure of career persistence and progress than the other measures that were static over the course of the study.

### Perceptions of subscale-related curricular elements

We conducted a survey where we asked our cohort to rank which elements of the curriculum they perceived as important in building their confidence in the subscale domains identified in this study. Although this method is purely descriptive, we believe it sheds insights on where to enhance our training programs. Curricular elements identified as building confidence in the surveyed competencies included grantwriting classes, rigor discussions, physician-scientist talks, role model and near-peer interviews, coaching, and whiteboard talks with peers. Our findings reinforce the value of coaching [44,45] and role models [46].

### Limitations

Our evaluation was conducted during the COVID-19 pandemic. All classes were virtual between spring 2020 and fall 2021 due to COVID-19 restrictions. The pandemic stressed academia, with higher academic costs for women [47,48]. It is interesting that in our cohort, women’s reported confidence increased despite the pandemic. While a full accounting is beyond the scope of this paper, in a separate survey the trainees were asked if they strongly disagreed (1) or strongly agreed (5) on a 5-point Likert scale with the statement: Changes to my home life due to the COVID-19 pandemic have greatly impacted my ability to work. The response of the full cohort was 3.0 (neutral) at both study timepoints. Neither men (*p* = 0.19, difference 0.39, 95% CI −0.21 to 0.98)) nor women (*p* = 0.26, difference −0.18, 95% CI −0.51 to 0.14) ranked the impact of COVID-19 on their work to change between the T2 and T1 timepoints. While not significant within gender groups, the slight decrease in women’s ranking of the burden of COVID-19 over time was significant (*p* = 0.048) in comparison to the difference over time in men’s ranking of the COVID-19 question. Although we did not detect a higher impact of COVID-19 on women as reported elsewhere, it is unclear whether that finding will be generalizable to the post-pandemic era.

Given that this is a single institution study, the generalizability of this survey tool to other training programs and settings remains to be determined. The survey of perceived confidence in professional, personal, and scientific competencies that we used has not been rigorously validated. Additionally, the EFA and outcome analyses were conducted with the same cohort so subscales derived may or may not generalize. We studied three physician-scientist training programs, primarily focused on preclinical research. Although some trainees engaged in both preclinical and clinical research, it is unclear if similar outcomes apply to programs limited to clinical research.

## Conclusions

During our pre- and postgraduate physician-scientist training programs, confidence in scientific, professional, and personal skills increased significantly in postgraduate trainees and at all training levels among women. This positive trend in women’s confidence during training may contribute to reducing gender gaps in persistence in academic medicine. Our findings aim to assist physician-scientist training program leaders as they evaluate their trainees and develop their curriculum.

## Methods

The 36 Likert-type survey items measuring self-rated confidence included 5 items from CRAI-12 [23]. Additional items were developed based on literature on barriers/facilitators identified by physician scientists and on the results of a programmatic needs assessment that we had previously conducted with 143 residents/fellow trainees equally divided between academic educational, clinical, or basic/translational research tracks at our institution. We retained the 11-point rating scale used in the CRAI. The final 36 items were assessed for face validity during cognitive interviews with MD-PhD alumni. Details on survey administration, exploratory factor analysis, design of mixed effects modeling, and the curricular survey are presented in the Supplement, Supplemental Methods.

## Supporting information

Khouja et al. supplementary material Khouja et al. supplementary material

## Supplementary material

The supplementary material for this article can be found at https://doi.org/10.1017/cts.2024.556.

## References

[1] Garrison HH , Ley TJ. Physician-scientists in the United States at 2020: trends and concerns. FASEB J. 2022;36(5):e22253. 35349197 10.1096/fj.202200327 PMC9314812

[2] Donowitz M , Germino G , Cominelli F , Anderson JM. The attrition of young physician-scientists: problems and potential solutions. Gastroenterology. 2007;132(2):477–480. 17258744 10.1053/j.gastro.2006.12.023

[3] Milewicz DM , Lorenz RG , Dermody TS , Brass LF , the National Association of MD-PhD Programs Executive Committee. Rescuing the physician-scientist workforce: the time for action is now. J Clin Invest. 2015;125(10):3742–3747. 26426074 10.1172/JCI84170 PMC4607120

[4] Salata RA , Geraci MW , Rockey DC , et al. U.S. physician-scientist workforce in the 21st century: recommendations to attract and sustain the pipeline. Acad Med. 2018;93(4):565–573. 28991849 10.1097/ACM.0000000000001950 PMC5882605

[5] Yeravdekar RC , Singh A. Physician-scientists: fixing the leaking pipeline - a scopingreview. Med Sci Educ. 2022;32(6):1413–1424. 36532399 10.1007/s40670-022-01658-y PMC9755418

[6] Ogdie A , Shah AA , Makris UE , et al. Barriers to and facilitators of a career as a physician-scientist among rheumatologists in the US. Arthritis Care Res (Hoboken). 2015;67(9):1191–1201. 25708626 10.1002/acr.22569 PMC4546916

[7] Keswani SG , Moles CM , Morowitz M , et al. The future of basic science in academic surgery: identifying barriers to success for surgeon-scientists. Ann Surg. 2017;265(6):1053–1059. 27643928 10.1097/SLA.0000000000002009 PMC5450912

[8] Daye D , Patel CB , Ahn J , Nguyen FT. Challenges and opportunities for reinvigorating the physician-scientist pipeline. J Clin Invest. 2015;125(3):883–887. 25689260 10.1172/JCI80933 PMC4362227

[9] Levey BA , Gentile NO , Jolly HP , Beaty HN , Levey GS. Comparing research activities of women and men faculty in departments of internal medicine. Acad Med. 1990;65(2):102–106. 2302295 10.1097/00001888-199002000-00011

[10] NIH. NIH data book, data by gender 2023).

[11] Ghosh-Choudhary S , Carleton N , Nouraie SM , Kliment CR , Steinman RA. Predoctoral MD-phD grants as indicators of future NIH funding success. JCI Insight. 2022;7(6):e155688. doi: 10.1172/jci.insight.155688. 35315356 PMC8986062

[12] Jones RD , Griffith KA , Ubel PA , Stewart A , Jagsi R. A mixed-methods investigation of the motivations, goals, and aspirations of male and female academic medical faculty. Acad Med. 2016;91(8):1089–1097. 27254012 10.1097/ACM.0000000000001244 PMC7357577

[13] Pololi L , Civian J , Brennan R , Dottolo A , Krupat E. Experiencing the culture of academic medicine: gender matters, a national study. J Gen Intern Med. 2013;28(2):201–207. 22936291 10.1007/s11606-012-2207-1 PMC3614142

[14] Vajapey S , Weber KL , Samora JB. Confidence gap between men and women in medicine: a systematic review. Current Orthopaedic Practice. 2020;31(5):494–502.

[15] Bakken LL , Sheridan J , Carnes M. Gender differences among physician-scientists in self-assessed abilities to perform clinical research. Acad Med. 2003;78(12):1281–1286. 14660433 10.1097/00001888-200312000-00018

[16] Gillen KM , Markowitz DM , Long P , Villegas-Estrada A , Chang E , Gupta A. National Institutes of Health funding gaps for principal investigators. JAMA Netw Open. 2023;6(9):e2331905. 37725378 10.1001/jamanetworkopen.2023.31905 PMC10509726

[17] Murphy M , Callander JK , Dohan D , Grandis JR. Women’s experiences of promotion and tenure in academic medicine and potential implications for gender disparities in career advancement: a qualitative analysis. JAMA Netw Open. 2021;4(9):e2125843. 34542616 10.1001/jamanetworkopen.2021.25843 PMC8453318

[18] Kwan JM , Gross CP. Improving support for physician scientists-mind the (funding) gap. JAMA Netw Open. 2023;6(9):e2332982. 37725379 10.1001/jamanetworkopen.2023.32982

[19] Jagsi R , DeCastro R , Griffith KA , et al. Similarities and differences in the career trajectories of male and female career development award recipients. Acad Med. 2011;86(11):1415–1421. 21952061 10.1097/ACM.0b013e3182305aa6

[20] Warner ET , Carapinha R , Weber GM , Hill EV , Reede JY. Faculty promotion and attrition: the importance of coauthor network reach at an academic medical center. J Gen Intern Med. 2016;31(1):60–67. 26173540 10.1007/s11606-015-3463-7 PMC4700018

[21] Kwan JM , Daye D , Schmidt ML , et al. Exploring intentions of physician-scientist trainees: factors influencing MD and MD/PhD interest in research careers. BMC Med Educ. 2017;17(1):115. 28697782 10.1186/s12909-017-0954-8 PMC5505137

[22] Mullikin EA , Bakken LL , Betz NE. Assessing research self-efficacy in physician-scientists: the clinical research appraisal inventory. J Career Assess. 2016;15(3):367–387.

[23] Robinson GF , Switzer GE , Cohen ED , et al. A shortened version of the clinical research appraisal inventory: CRAI-12. Acad Med. 2013;88(9):1340–1345. 23886999 10.1097/ACM.0b013e31829e75e5 PMC3758379

[24] Steinman RA , Proulx CN , Levine AS. The highly structured physician scientist training program (PSTP) for medical students at the University of Pittsburgh. Acad Med. 2020;95(9):1373–1381. 32079926 10.1097/ACM.0000000000003197 PMC7447180

[25] Shah AM , Rao RJ. Promoting female physician-scientists: perspectives from a unique learning environment. J Clin Transl Sci. 2023;7(1):e87. 37125069 10.1017/cts.2023.38 PMC10130830

[26] Harris PA , Taylor R , Minor BL , et al. The REDCap consortium: building an international community of software platform partners. J Biomed Inform. 2019;95:103208. 31078660 10.1016/j.jbi.2019.103208 PMC7254481

[27] Harris PA , Taylor R , Thielke R , Payne J , Gonzalez N , Conde JG. Research electronic data capture (REDCap)--a metadata-driven methodology and workflow process for providing translational research informatics support. J Biomed Inform. 2009;42(2):377–381. 18929686 10.1016/j.jbi.2008.08.010 PMC2700030

[28] Robinson GF , Switzer GE , Cohen ED , et al. Shortening the work preference inventory for use with physician scientists: WPI-10. Clin Transl Sci. 2014;7(4):324–328. 24405561 10.1111/cts.12132 PMC4090299

[29] Dolan ED , Mohr D , Lempa M , et al. Using a single item to measure burnout in primary care staff: a psychometric evaluation. J Gen Intern Med. 2015;30(5):582–587. 25451989 10.1007/s11606-014-3112-6 PMC4395610

[30] Diener E , Emmons RA , Larsen RJ , Griffin S. The satisfaction with life scale. J Pers Assess. 1985;49(1):71–75. 16367493 10.1207/s15327752jpa4901_13

[31] Duckworth AL , Quinn PD. Development and validation of the short grit scale (grit-s). J Pers Assess. 2009;91(2):166–174. 19205937 10.1080/00223890802634290

[32] Winkel AF , Chang LY , McGlone P , Gillespie C , Triola M. SMARTer goalsetting: a pilot innovation for coaches during the transition to residency. Acad Med. 2023;98(5):585–589. 36652456 10.1097/ACM.0000000000005153

[33] Epstein N , Fischer MR. Academic career intentions in the life sciences: can research self-efficacy beliefs explain low numbers of aspiring physician and female scientists? PLoS One. 2017;12(9):e0184543. 28910334 10.1371/journal.pone.0184543 PMC5598975

[34] Lips H. The gender gap in possible selves: divergence of academic self-views among high school and university students. Sex Roles. 2004;50(5/6):357–371.

[35] Klassen RM , Klassen JRL. Self-efficacy beliefs of medical students: a critical review. Perspect Med Educ. 2018;7(2):76–82. 29484552 10.1007/s40037-018-0411-3 PMC5889382

[36] Bierer SB , Prayson RA , Dannefer EF. Association of research self-efficacy with medical student career interests, specialization, and scholarship: a case study. Adv Health Sci Educ Theory Pract. 2015;20(2):339–354. 25037264 10.1007/s10459-014-9531-7

[37] Lipira L , Jeffe DB , Krauss M , et al. Evaluation of clinical research training programs using the clinical research appraisal inventory. Clin Transl Sci. 2010;3(5):243–248. 21442017 10.1111/j.1752-8062.2010.00229.x PMC3062999

[38] DiBiase RM , Beach MC , Carrese JA , et al. A medical student scholarly concentrations program: scholarly self-efficacy and impact on future research activities. Med Educ Online. 2020;25(1):1786210. 32589550 10.1080/10872981.2020.1786210 PMC7482758

[39] Isaac C , Kaatz A , Lee B , Carnes M. An educational intervention designed to increase women’s leadership self-efficacy. CBE Life Sci Educ. 2012;11(3):307–322. 22949427 10.1187/cbe.12-02-0022 PMC3433303

[40] Betz N , Schifano R. Evaluation of an intervention to incresae realistic self-efficacy and interests in college women. J Vocat Behav. 2000;56(1):35–52.

[41] Kruger J , Dunning D. Unskilled and unaware of it: how difficulties in recognizing one’s own incompetence lead to inflated self-assessments. J Pers Soc Psychol. 1999;77(6):1121–1134. 10626367 10.1037//0022-3514.77.6.1121

[42] Akabas MH , Brass LF. The national MD-PhD program outcomes study: outcomes variation by sex, race, and ethnicity. JCI Insight. 2019;4(19):e133010. doi: 10.1172/jci.insight.133010. 31578303 PMC6795407

[43] Neroni J , Meijs C , Kirschner P , Xu K , de Groot R. Academic self-efficacy, self-esteem, and grit in higher online education: consistency of interests predicts academic success. Soc Psychol Educ. 2022;5(4):951–975.

[44] Deiorio NM , Carney PA , Kahl LE , Bonura EM , Juve AM. Coaching: a new model for academic and career achievement. Med Educ Online. 2016;21(1):33480. 27914193 10.3402/meo.v21.33480 PMC5136126

[45] Sabatine J , Wendell S , Paper presented at the Institute of Coaching in Leadership and Healthcare Conference, Boston, Massachusetts, 2017.

[46] Bakken LL. Who are physician-scientists’ role models? Gender makes a difference. Acad Med. 2005;80(5):502–506. 15851466 10.1097/00001888-200505000-00020

[47] Ellinas EH , Ark TK , Kaljo K , Quinn KG , Krier CR , Farkas AH. Winners and losers in academic productivity during the COVID-19 pandemic: is the gender gap widening for faculty? J Womens Health (Larchmt). 2022;31(4):487–494. 34935469 10.1089/jwh.2021.0321

[48] Weinreich HM , Kotini-Shah P , Man B , et al. Work-life balance and academic productivity among College of Medicine faculty during the evolution of the COVID-19pandemic: the new normal. Womens Health Rep (New Rochelle). 2023;4:367–380. 37476606 10.1089/whr.2023.0007 PMC10354727

