# Supplementary material for: Changes in self-confidence in professional, personal, and scientific skills by gender during physician-scientist training at the University of Pittsburgh
Source: J Clin Transl Sci. 2024 Jun 5;8(1):e106. doi: 10.1017/cts.2024.556 (PMC11617090; doi:10.1017/cts.2024.556)
Supplement: Khouja et al. supplementary material [file S2059866124005569sup001.docx]

**Supplemental Digital Content**

1. Table 1. Characteristics of participants
2. Figure 1. Mean responses to confidence ranking items in total, by career stage and by survey time.
3. Figure 2. Average level of confidence in ability to perform each skill listed in the survey.
4. Table 2. Differences between males and females in mean scores across research confidence skills subscales at initial assessment and at followup
5. Table 3*.* Mean scores of self- confidence subscales by career level (BWF Fellows & MSTP/PSTP) and gender at time 1 and time 2.
6. Table 4. Changes in grit, motivation, satisfaction, burnout by gender
7. Figure 3. Motivation, Grit, Satisfaction, Burnout among responding MSTP/PSTP.
8. Figure 4. Motivation, Grit, Satisfaction, Burnout among responding BWF Fellows.
9. Table5A, B, C, D. Curriculum survey, rankings of curricular elements by perceived impact on confidence subscales, and list of curriculum details.
10. Figure 5. Visual Abstract
11. Supplemental methods

Table 1. Cohort characteristics (N=100)

|  | **N** | **%** |
| --- | --- | --- |
| **Gender** |  |  |
| Male | 39 | 39 |
| Female | 61 | 61 |
| **Ethnicity** |  |  |
| Not Hispanic or Latino | 88 | 88 |
| Hispanic or Latino | 9 | 9 |
| Prefer not to answer | 2 | 2 |
| Missing | 1 | 1 |
| **Race** |  |  |
| American Indian or Alaska Native | 0 | 0 |
| Asian | 40 | 40 |
| Black or African American | 5 | 5 |
| Native Hawaiian or other Pacific Islander | 0 | 0 |
| White/Caucasian | 53 | 53 |
| Prefer not to answer | 2 | 2 |
| **Program** |  |  |
| BWF | 18 | 18 |
| MSTP/PSTP | 82 | 82 |

***Suppl. Table 1. Characteristics of participants.*** *All participants responded to both the initial and followup surveys.*

**Suppl. Figure 1.**  **Mean responses to confidence ranking items in total, by career stage and by survey time.** Mean responses by 101 trainees (83 MSTP/PSTP, 18 BWF Fellows) to each of 36 ranking items are shown. Each dot represents an item on the survey. Bars show mean of indicated group/time to all 36 ranking items. ****p<0.0001 by non-paired t test.

*MSTP, Medical Scientist Training Program; PSTP, Physician Scientist Training Program (medical student); BWF,*

*Burroughs Wellcome Foundation (BWF physician scientist incubator for residents and fellows).*

**Suppl. Figure 2 (Following pages). Average level of confidence in ability to perform each skill listed in the survey. A.** All participants, **B.** All males, **C.** All females, **D.** BWF Fellows, **E.** MSTP/PSTP medical students at initial (blue) and ~2 year followup (orange)

*MSTP, Medical Scientist Training Program; PSTP, Physician Scientist Training Program (medical student); BWF,*

*Burroughs Wellcome Foundation (BWF physician scientist incubator for residents and fellows).*

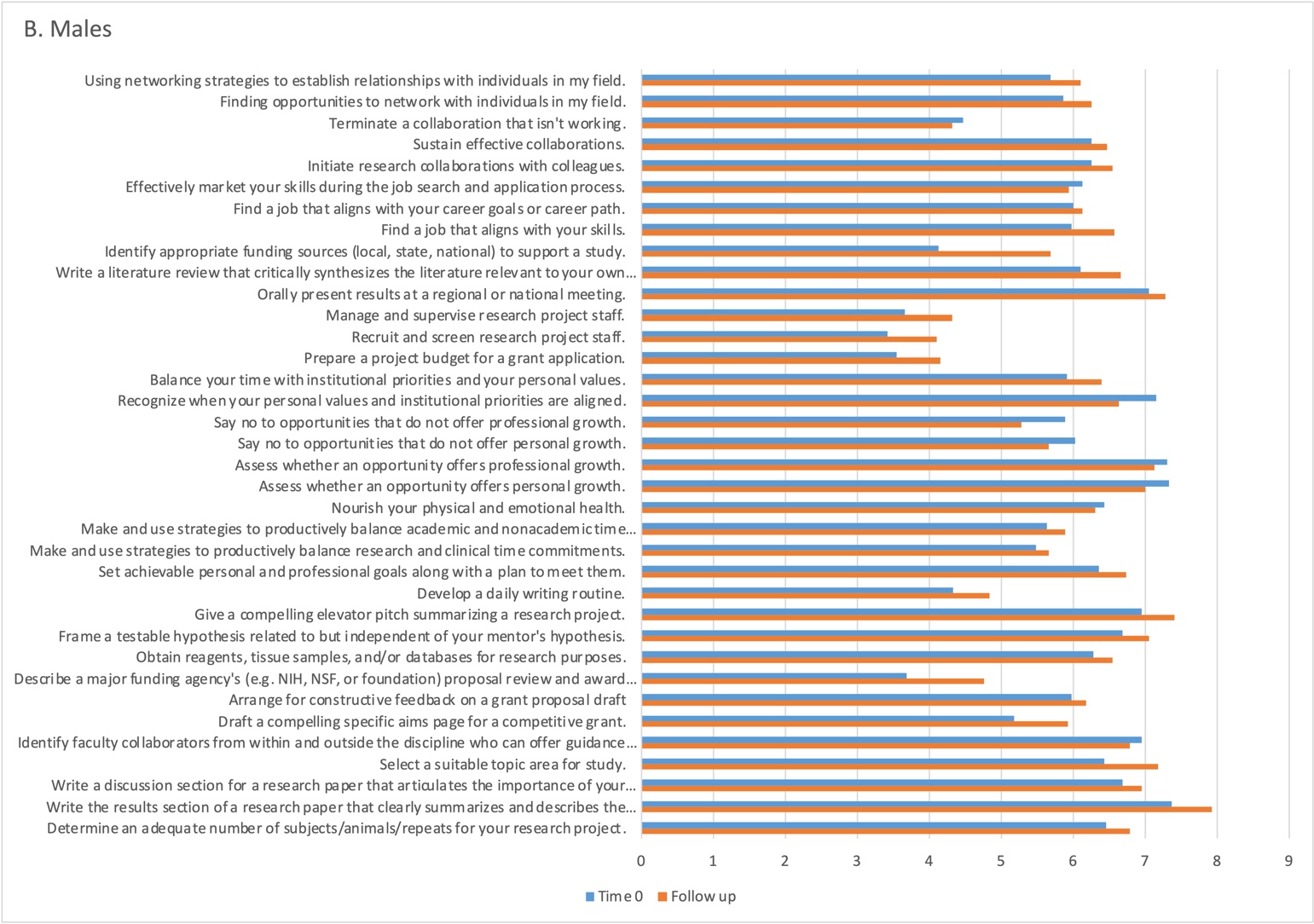


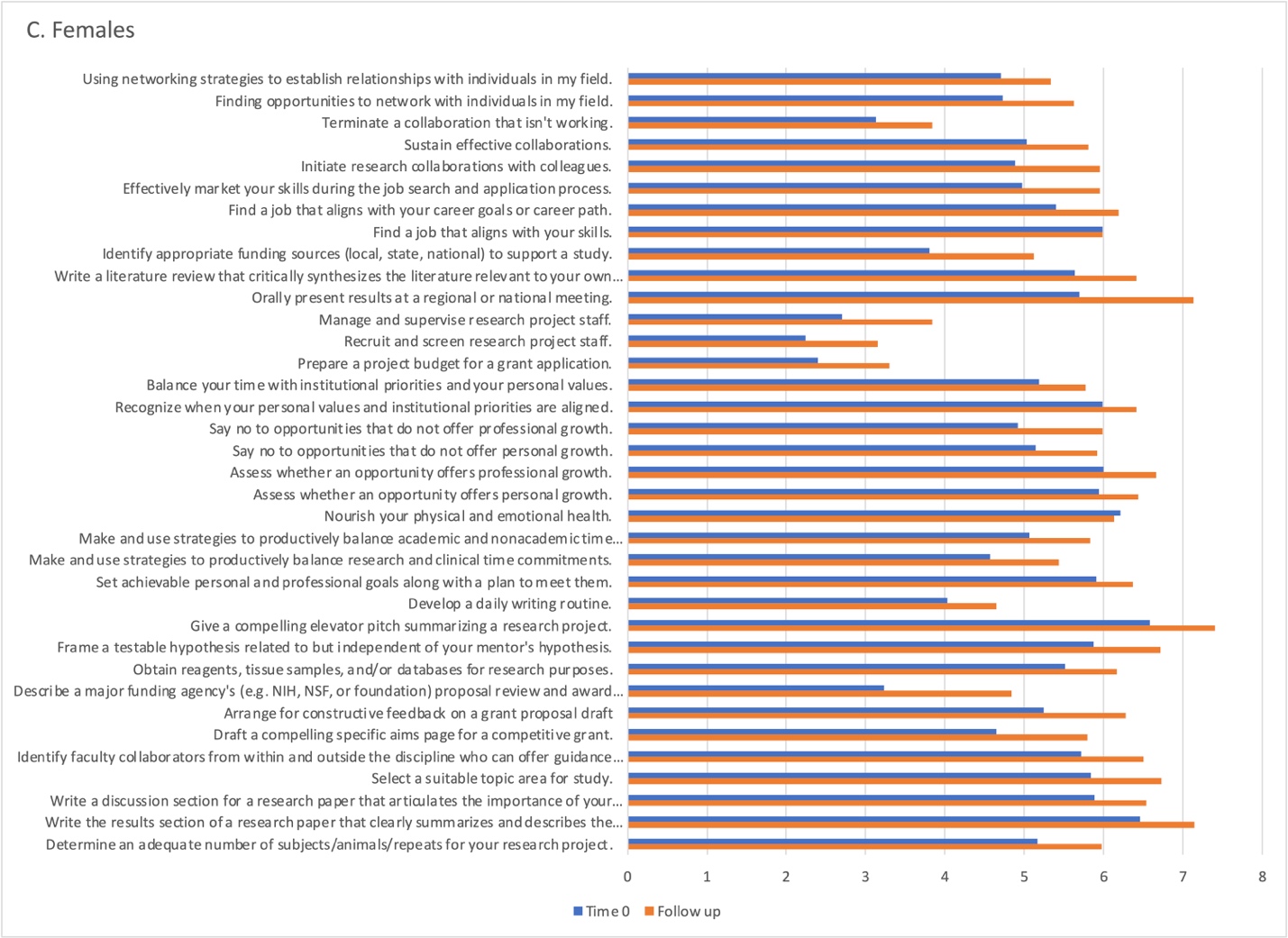

|  | Males | | | Females | | | Difference (Males- Females) | SD | p-value |
| --- | --- | --- | --- | --- | --- | --- | --- | --- | --- |
| Subscale | N | Mean | SD | N | Mean | SD |  |  |  |
| Initial Assessment, Time 1 | | | | | | | | | |
| Career sustainability | 38 | 6.0 | 1.9 | 59 | 4.8 | 1.9 | 1.1 | 1.9 | 0.0056 |
| Science productivity | 38 | 6.5 | 1.4 | 60 | 5.7 | 1.5 | 0.8 | 1.5 | 0.0076 |
| Grant management | 38 | 4.2 | 2.2 | 60 | 3.5 | 1.8 | 0.7 | 2.0 | 0.0788 |
| Goal setting | 39 | 5.7 | 1.7 | 61 | 5.2 | 1.9 | 0.5 | 1.8 | 0.1769 |
| Goal alignment | 38 | 6.2 | 1.4 | 59 | 5.3 | 1.7 | 0.9 | 1.7 | 0.0053 |
| Follow up, Time 2 | | | | | | | | | |
| Career sustainability | 38 | 6.2 | 1.3 | 59 | 5.7 | 1.8 | 0.4 | 1.6 | 0.1854 |
| Science productivity | 38 | 6.9 | 1.1 | 60 | 6.5 | 1.5 | 0.4 | 1.3 | 0.1986 |
| Grant management | 38 | 5.0 | 1.6 | 60 | 4.6 | 1.9 | 0.4 | 1.8 | 0.3409 |
| Goal setting | 39 | 5.9 | 1.6 | 61 | 5.7 | 1.7 | 0.2 | 1.7 | 0.5472 |
| Goal alignment | 38 | 6.0 | 1.5 | 59 | 6.0 | 1.7 | 0.0 | 1.6 | 0.9702 |

P-values were derived from an independent sample t-test

Participants who completed research confidence skill items included in each subscale at both time 1 and time 2 were included in the analysis

**Suppl. Table 2. Differences between males and females in mean scores across research confidence skills subscales at initial assessment and at followup.** *MSTP, Medical Scientist Training Program; PSTP, Physician Scientist Training Program (medical student); BWF,*

*Burroughs Wellcome Foundation (BWF physician scientist incubator for residents and fellows).*

| Program | **BWF Fellows** | | | | | | | | **PSTP/MSTP** | | | | | | | |
| --- | --- | --- | --- | --- | --- | --- | --- | --- | --- | --- | --- | --- | --- | --- | --- | --- |
| Subscale | N | Time 1  initial | | Time 2 Follow up | | time2 minus time 1 | SD | p-value | N | Time 1 initial | | Time 2 Follow up | | time2minus time1 | SD | p-value |
|  |  | Mean | SD | Mean | SD |  |  |  |  | Mean | SD | Mean | SD |  |  |  |
| **Males** | | | | | | | | | | | | | | | | |
| Career sustainability | 6 | **5.5** | 2.0 | **7.0** | 1.3 | 1.5 | 1.2 | 0.023 | 32 | **6.0** | 1.9 | **6.0** | 1.3 | 0.0 | 1.6 | 0.980 |
| Science productivity | 6 | **5.8** | 1.7 | **7.3** | 0.7 | 1.5 | 1.2 | 0.027 | 32 | **6.6** | 1.4 | **6.8** | 1.1 | 0.1 | 1.1 | 0.486 |
| Grant management | 6 | **4.5** | 2.3 | **6.3** | 1.8 | 1.8 | 1.1 | 0.012 | 32 | **4.2** | 2.2 | **4.8** | 1.5 | 0.6 | 1.8 | 0.067 |
| Goal setting | 6 | **4.9** | 1.0 | **6.3** | 1.4 | 1.4 | 1.0 | 0.017 | 33 | **5.8** | 1.7 | **5.8** | 1.7 | 0.0 | 1.5 | 0.921 |
| Goal Alignment | 6 | **5.6** | 1.8 | **6.9** | 0.8 | 1.3 | 1.7 | 0.120 | 32 | **6.4** | 1.4 | **5.8** | 1.6 | -0.5 | 1.4 | 0.039 |
| **Females** | | | | | | | | | | | | | | | | |
| Career sustainability | 12 | **4.9** | 1.4 | **6.9** | 1.1 | 2.0 | 1.4 | <0.001 | 47 | **4.8** | 2.0 | **5.4** | 1.8 | 0.6 | 1.5 | 0.008 |
| Science productivity | 12 | **5.1** | 1.3 | **7.4** | 1.4 | 2.3 | 1.4 | <0.001 | 48 | **5.8** | 1.5 | **6.3** | 1.4 | 0.5 | 1.1 | 0.006 |
| Grant management | 12 | **3.3** | 1.4 | **6.0** | 1.4 | 2.7 | 1.8 | <0.001 | 48 | **3.5** | 1.9 | **4.3** | 1.8 | 0.8 | 1.6 | 0.002 |
| Goal setting | 12 | **5.4** | 1.6 | **7.0** | 1.3 | 1.6 | 1.5 | 0.003 | 49 | **5.1** | 2.0 | **5.4** | 1.6 | 0.3 | 1.7 | 0.223 |
| Goal Alignment | 12 | **5.8** | 1.5 | **6.5** | 2.0 | 0.8 | 1.2 | 0.056 | 47 | **5.2** | 1.7 | **5.9** | 1.6 | 0.7 | 1.8 | 0.010 |

**Suppl. Table 3*.* Mean scores of self- confidence subscales by career level (BWF Fellows & MSTP/PSTP) and gender at time 1 and time 2.** *p-values were derived from a paired t-test. Participants who completed research confidence skill items included in each subscale at time 1and time 2 were included in the analysis. MSTP, Medical Scientist Training Program; PSTP, Physician Scientist Training Program (medical student); BWF, Burroughs Wellcome Foundation (BWF physician scientist incubator for residents and fellows).*

|  | Males | | | Females | | | Difference (Males- Females) | 95% CI | p-value |
| --- | --- | --- | --- | --- | --- | --- | --- | --- | --- |
| Subscale | N | Mean | SD | N | Mean | SD |  |  |  |
| Initial (Time 1) | | | | | | | | | |
| Grit | 37 | **3.48** | 0.59 | 56 | **3.81** | 0.55 | -0.33 | -0.56 to -0.10 | 0.006 |
| Extrinsic Motivation | 44 | **2.48** | 0.63 | 65 | **2.51** | 0.48 | -0.02 | -0.18 to 0.23 | 0.831 |
| Intrinsic Motivation | 44 | **3.36** | 0.45 | 65 | **3.20** | 0.46 | 0.16 | \| -0.01 to 0.33 \| \| --- \| | 0.069 |
| Satisfaction | 38 | **4.95** | 1.04 | 55 | **5.16** | 1.25 | -0.21 | -0.70 to 0.29 | 0.978 |
| Burnout | 40 | **1.85** | 0.62 | 60 | **2.00** | 0.71 | -0.15 | -0.42 to 0.12 | 0.281 |
| Follow up (Time 2) | | | | | | | | | |
| Grit | 37 | **3.51** | 0.55 | 56 | **3.79** | 0.45 | -0.28 | -0.49 to -0.07 | 0.009 |
| Extrinsic Motivation | 44 | **2.38** | 0.51 | 65 | **2.51** | 0.55 | -0.12 | -0.32 to 0.08 | 0.234 |
| Intrinsic Motivation | 44 | **3.32** | 0.49 | 64 | **3.23** | 0.43 | 0.09 | -0.07 to 0.27 | 0.275 |
| Satisfaction | 38 | **5.01** | 1.14 | 55 | **5.21** | 1.22 | -0.20 | -0.70 to 0.30 | 0.425 |
| Burnout | 40 | **2.15** | 0.86 | 60 | **2.10** | 0.72 | 0.05 | -0.27 to 0.37 | 0.756 |

P-values were derived from unpaired t tests performed separately for each gender comparison for a given subscale and time.

Participants who completed research confidence skill items included in each subscale at time 1 and follow up were included in the analysis

**Suppl. Table 4. Changes in grit, motivation, satisfaction, burnout by gender.** *MSTP, Medical Scientist Training Program; PSTP, Physician Scientist Training Program (medical student); BWF,*

*Burroughs Wellcome Foundation (BWF physician scientist incubator for residents and fellows). SD. Standard deviation.*

**Suppl. Figure 3. Motivation, Grit, Satisfaction, Burnout among responding MSTP/PSTP.**

Motivation responses (5 point scale) divided into extrinsic (extr) and intrinsic (intr) motivating factors; Responses to motivation, grit (5 point scale), satisfaction (7 point scale) and burnout (5 point scale) are shown by gender. Each dot is a respondent. *MSTP, Medical Scientist Training Program; PSTP, Physician Scientist Training Program (medical student).*

**
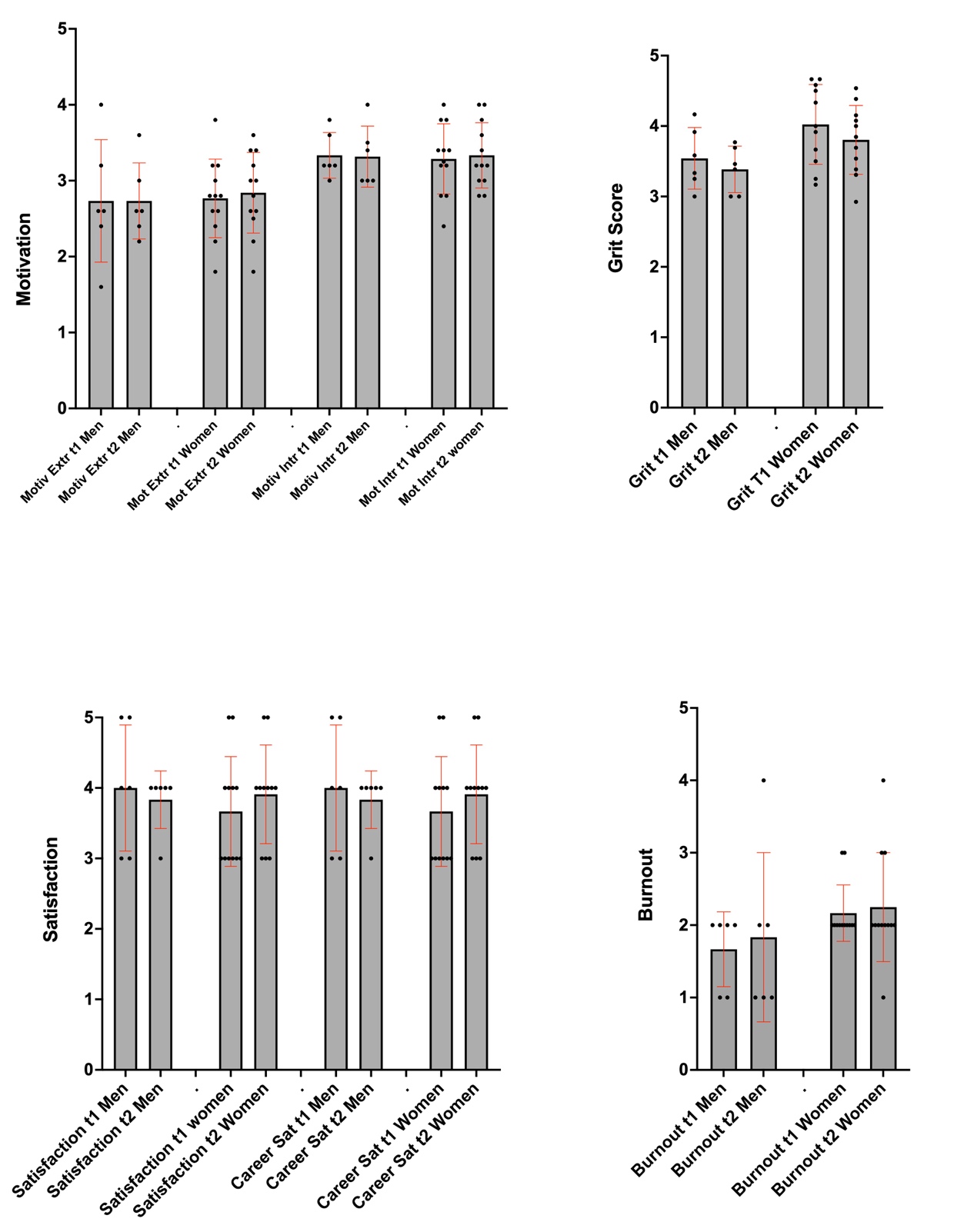
**

**Suppl. Figure 4. Motivation, Grit, Satisfaction, Burnout among responding BWF**

**Fellows.** Motivation responses (5 point scale) divided into extrinsic (extr) and intrinsic (intr) motivating factors; Responses to motivation, grit (5 point scale), satisfaction (7 point scale) and burnout (5 point scale) are shown by gender. Each dot is a respondent. *BWF,*

*Burroughs Wellcome Foundation (BWF physician scientist incubator for residents and fellows).*

**Suppl. Table 5.**

1. **Curriculum ranking survey**

Part 1, Instructions and Response Grid

Thank you for participating in this survey!

The BWF, MSTP, and PSTP programs are interested in seeing how different aspects of the program curriculum impact confidence in areas related to success in physician scientist careers. This survey is expected to take approximately 5-10 minutes to complete. On the next page, you'll be asked 5 questions. Each question will ask you to select up to 3 aspects of physician scientist training curriculum that positively impacted your confidence in one of 5 focus areas. You may choose the same curriculum component(s) for multiple focus areas. After each checkbox question, there is an optional comment box where you may explain how the curriculum components impacted that focus area.

The 5 focus areas are: Career sustainability: refers to your ability to successfully network and market yourself, develop and sustain collaborations that support your career, and find jobs that align with career goals and skills. Science productivity: refers to your ability to make hypotheses, design a research study, produce effective publications and presentations detailing research findings, and get scientific advice. Grant management: refers to your ability to write and submit research grants and recruit, hire, manage, and supervise research staff. Healthy goal setting: refers to your ability to set achievable personal and professional goals, nourish physical and emotional health, and balance clinical, research, and personal time commitments. Goal alignment: refers to your ability to say "no" to opportunities that do not offer personal and/or professional growth and recognize when your values and institutional priorities are aligned. Your participation in this survey will help us better understand what aspects of the curriculum are particularly helpful for growing physician scientist confidence in areas related to research success. No identifiable information will be shared with your responses, which will be summarized for program leadership.

|  | Career Sustainability | Science Productivity | Grant  Management | Healthy  Goal  Setting | Goal  Alignment |
| --- | --- | --- | --- | --- | --- |
| Career coaching (group workshops and/or individual) |  |  |  |  |  |
| Case reports on career challenges |  |  |  |  |  |
| Grant writing classes |  |  |  |  |  |
| Imposter syndrome class |  |  |  |  |  |
| Journal clubs |  |  |  |  |  |
| Peer interactions in class |  |  |  |  |  |
| Presentations by physician scientists |  |  |  |  |  |
| Professional development courses/activities |  |  |  |  |  |
| Research basis of medical knowledge 3 semesters |  |  |  |  |  |
| Rigor/responsibility classes |  |  |  |  |  |
| Role model interview exercises and/or Mentor mapping exercise/Near peer interviews |  |  |  |  |  |
| Self-promotion activities/negotiate for position class |  |  |  |  |  |
| Statistics classes |  |  |  |  |  |
| Whiteboard presentations |  |  |  |  |  |
| Workshop sessions |  |  |  |  |  |
| Other, please specify: |  |  |  |  |  |

Please let us know how the curriculum components you selected above positively impacted your confidence surrounding each of the subscales.

| **Subscale** | **Course/class activity in curriculum ranked as most relevant** |
| --- | --- |
| Career Sustainability | Professional development classes |
| Science Productivity | Whiteboard presentations, Rigor/reproducibility classes |
| Grant Management | Grant writing course/classes |
| Goal Setting | Professional coaching, peer interactions in class |
| Goal Alignment | Professional coaching, peer interactions in class |

**Suppl. Table 5B. Curricular elements that were highly cited (i.e. among the top 4 contributory training activities) for each listed subscale by all 3 of the surveyed cohorts (BWF, MSTP, PSTP).**

**Suppl. Table 5C**. Detail, top 4 curriculum elements by training program identified for each subscale.

Courses/activities taught in the training programs

| Course/Activity | BWF | MSTP | PSTP |
| --- | --- | --- | --- |
| Concierge (fiscal support of time saving/wellness activities) | x |  |  |
| Professional Career Coaches (resiliency, strategic planning, etc) | x | x | x |
| Career Advisor (non-mentor) or Career Development Committee | x | x | x |
| Formal informational Interviews with Near Peers/role models | x | x | x |
| Multi-clinical specialty or multidiscipline in classes together | x | x | x |
| Case reports on career challenges | x | x | x |
| Imposter syndrome class | x | x | x |
| Structured peer interactions in class | x | x | x |
| Regular whiteboard work-in-progress talks | x | x | x |
| Writing groups-evaluate papers or grants (grant class) by peers | x | x | x |
| Grantwriting classes, multiple topics and mock study sections | classes | course | course |
| Biostatistics | x | x | optional |
| Topical Workshops | conference | x | x |
| Rigor/responsibility classes (multiple topics) | x | x | x |
| Professional development classes (multiple shared topics e.g. Self promotion, reponding to reviewer feedback, perfecting your biosketch, limits of experimental plans, powering science, sources of error , decision theory, elevator pitches etc) | topics | 3 courses | 2 courses |
| Personal Identity Essays | x |  | x |
| Research basis of medical knowledge, including journal clubs |  | 3 courses | 3 courses |
| Ethics and responsible conduct of research | classes | course | classes |
| Unique BWF Topics: (e.g. how to obtain an optimal faculty position, budgeting, laboratory leadership, translational -omics best practices,  Empowering clinical and research teams to work together) | x |  |  |
| Unique MSTP (grantwriting course is focused on F30 grants only ) |  | x |  |
| Program wide workshops and annual retreat |  | x | x |

**Suppl. Table 5D. List of curricular elements shared by or specific to each training program.**

**
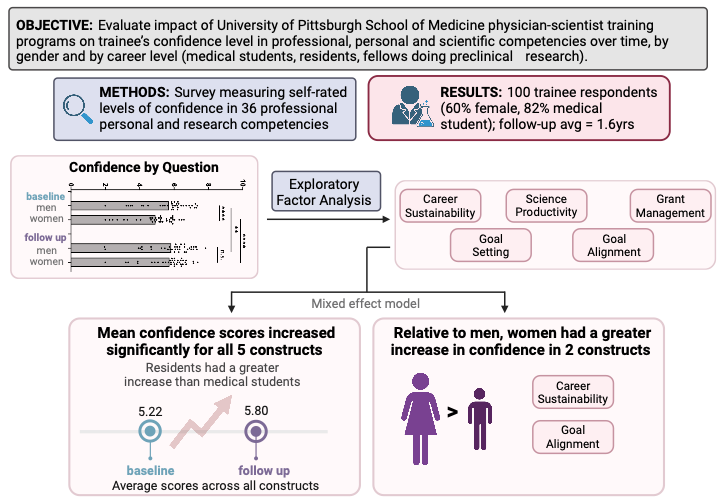
**

**eFigure 5.** **Visual Abstract (Created with** [**BioRender.com**](https://biorender.com/)**)**

**Suppl. Methods**

Participants comprised medical students in physician scientist training programs (MSTP and PSTP) as well as in supported residents and fellows training in pre-clinical investigation in our BWF Incubator.

A survey including 36 Likert-type items measuring self-rated confidence in discrete professional, personal and scientific competencies was administered. Survey items included 5 items from CRAI-12^23^ (broadening one item (“*Determine an adequate number of subjects for your research project”* to *“adequate number of subjects/animals/repeats”)*. Additional items were developed based on literature on barriers/facilitators identified by physician scientists and on the results of a programmatic needs assessment that we had previously conducted with 143 resident/fellow trainees equally divided between academic educational, clinical, or basic/translational research tracks at our institution. We retained the original 0 (no confidence) to 10 (total confidence) rating scale used in the CRAI. The final 36-items were assessed for face validity during cognitive interviews with MD-PhD alumni. The confidence scale was included in a larger survey instrument that included validated scales for motivation (intrinsic and extrinsic motivational factors) ^27^, burnout ^28^, satisfaction ^29^ and grit. ^30^ Descriptive statistics of changes in these parameters by gender were compared for the study cohort and by training level. Demographic questions included the participant’s age; an exploratory question on how their mentors met their expectations and on the impact of COVID-19 on their work were included in the questionnaire.

Prior to analysis of survey results, we conducted an exploratory factor analysis (EFA) in Stata 17.0 [StataCorp] to identify thematic subscales based on the 36 self-confidence items included in the survey. EFA was conducted using the responses of the first 151 participants who completed the confidence self-assessment as part of their time 1 assessment. An eigenvalue minimum cut-off of >1.00 was used to determine the number of factors we would retain. The results of the EFA suggested a 5-factor solution, explaining 87% of the total variance. We used an oblique promax rotation to determine item factor loadings for each of the 5 factors. All of the 36 items loaded cleanly onto the 5 factors using a criterion of >0.40 for factor loadings except for 3 items (i.e. *Obtain reagents, tissue samples, and/or databases for research purposes;* *Balance your time with institutional priorities and your personal values;* and *Orally present results at a regional or national meeting*). After reviewing the 3 items among the study team, it was determined that they were conceptually relevant for measuring the objectives of the program, and the items were placed on the factor where they had the greatest inter-item correlations. The 5 subscales arising from EFA will be referred to by the corresponding subscales hereafter: *Career Sustainability; Science Productivity; Grant Management; Goal Setting; and Goal Alignment.* Cronbach’s alpha was calculated for each subscale to confirm sufficient internal consistency and ranged from 0.85 to 0.94. Respondent scores were then averaged for each subscale identified.

Our analysis focuses on participants who completed both an initial (i.e. time 1) and followup (i.e. time 2) survey on confidence between 2020 and 2023. Ineligible individuals included 26 who had just begun their programs when the study concluded, and 4 individuals on extended multi-year leave. 206 eligible individuals were contacted, of whom 173 responded and 170 consented. 68 completed only 1 of the 2 surveys; 102 completed both surveys of whom 2 were excluded either because of preferring not to list gender or changing an answer on the initial survey while logged in to complete the followup survey. The final pool of 100 respondents who were analyzed for the study represented 57% of all eligible participants who consented. Nonrespondents did not differ from the 100 respondents analyzed in age (p=0.68) or gender (58% v 61% female). The nonrespondents who completed only the initial survey had similar confidence scores to the responders in the study (5.44 to 5.49, p=0.84). Based on those similarities, the likelihood of selection bias shifting results between study enrollees and the entire eligible pool is low. The time 1 survey was generally completed in late summer and time 2 in early spring the following year. The average time between the time 1 initial and time 2 followup survey was 1.6 years with a range of 0.8-3.9 years (this range includes 2 individuals who had to do a clinical year between the baseline survey and the start of Incubator classes/research; excluding them the range was 0.8-2.8 years). There was no correlation between the lag between matriculation and time 1 and respondent’s scoring of confidence at time 1 (r^2^=0.028 on linear regression). Only those who consented and completed both timepoints for self-rated confidence were retained in the analysis. This study was approved by the University of Pittsburgh IRB under expedited review.

We describe mean scores across subscales classified by gender and by career level. Paired t-tests were used to test significant changes in mean scores overall, by gender and by career level. Independent t-tests were used to test mean score differences by gender.

A mixed effects linear model with a random intercept and a difference in differences (DID) design was used to assess the differential impact of the programming by gender and by career level. We ran five models with each of the five subscales as the outcome of interest. In each model, we included an interaction term between gender and time to evaluate the differential impact by gender. Similarly, to assess the differential effect by career level, we ran five mixed effect linear models with an interaction term between career level (MSTP/PSTP vs BWF Fellows) and time.  Due to similar career stage and 4 shared physician scientist training classes that MSTP and PSTP students took, the MSTP and PSTP results were grouped together to allow for a more parsimonious model. This interaction term is the DID estimator defined as the difference in average scores in one group before and after exposure to our program minus the difference in average outcome in the second group before and after exposure to our program.  Each model controlled for the relative subscale initial score.  Bootstrapping method with 100 replications was used to estimate confidence intervals. We report predicted probabilities using the *margins* command in STATA. All data management and analysis were conducted using SAS version 9.4 (SAS Institute) and STATA version 17.0 (StataCorp).

To explore which components of the curriculum the trainees associated with their self-confidence, we created a curriculum survey (see Appendix for details). This survey included components of their curriculum that relate to the confidence subscales identified through the EFA (career sustainability, science productivity, grant management, goal setting and goal alignment). This voluntary survey was sent out to participants included in the analysis with multiple reminders between November/December 2022 and June/July 2023. Participants were instructed to rank three curriculum components that they perceived as pertinent to building confidence in each subscale. Three of the 15 activities queried (*Presentations by physician scientists, journal clubs, Research basis of Medical Knowledge course)* were distinct to the medical students and so were not included in the BWF Fellow survey whereas 12 courses/classes/activities were common to all 3 programs.
